# A qualitative study of perspectives on the acceptability and feasibility of “virtual home visits” for asthma

**DOI:** 10.1186/s12889-023-17485-8

**Published:** 2023-12-20

**Authors:** Mary E. Crocker, James W. Stout

**Affiliations:** 1 Seattle Children’s Research Institute, Seattle Children’s Hospital, Seattle, WA USA; 2 Division of Pulmonary & Sleep Medicine, Department of Pediatrics, University of Washington, Seattle, WA USA; 3 Department of Pediatrics, School of Medicine, University of Washington, Seattle, WA USA; 4 Department of Health Systems and Population Health, School of Public Health, University of Washington, Seattle, WA USA

**Keywords:** Asthma, Community health worker, Videoconferencing, Home visit, Qualitative, Indoor allergens

## Abstract

**Background:**

Asthma home-visit programs delivered by community health workers (CHWs) are an effective way to improve asthma outcomes and cost of care, through performing home environmental inspections, delivering education and hands-on demonstrations, and providing personalized behavior change support. During the COVID-19 pandemic, many in-person asthma CHW programs have been adapted to be delivered virtually, but it is unclear whether this is acceptable or feasible for clients with asthma. This qualitative study sought to identify perspectives of prior clients of the Public Health–Seattle & King County Asthma Program on acceptability and feasibility of a hypothetical virtual asthma program.

**Methods:**

We performed semi-structured interviews with participants speaking English, Spanish, and Somali. An *a priori* codebook was developed based on the Theoretical Framework of Acceptability and was revised iteratively during coding. Intra-rater reliability was established, and thematic analysis was used to determine major themes.

**Results:**

A total of 19 individuals participated (9 speaking English, 8 Spanish, and 2 Somali). Krippendorf’s alpha was 0.848, indicating high intra-rater reliability. Our results demonstrated that many participants felt positively about the prospect of completing the program virtually, but they also expected a variety of challenges, the most important of which were lack of engagement with the CHW and lack of confidence in the accuracy of a virtual home inspection. Participants also varied widely in their comfort level with videoconferencing platforms and their access to adequate internet connectivity.

**Conclusions:**

Acceptability and feasibility of virtual programming varies widely between participants, indicating that there may be no “one-size-fits-all” approach. We present several recommendations for adapting in-person asthma home visit programs to a virtual format, including considering a hybrid approach to delivery, making concerted efforts to build rapport when using videoconferencing, and deliberately evaluating the effectiveness of new adaptations, especially if a virtual environmental assessment is attempted.

**Supplementary Information:**

The online version contains supplementary material available at 10.1186/s12889-023-17485-8.

## Background

Asthma is a significant health problem in the state of Washington, where it affects over 600,000 adults [1] and 104,000 children annually [2]. Indoor environmental triggers such as poor air quality (air pollution, second-hand smoke, chemical irritants) and allergens (e.g. dust mites, mold, pollen, pests) are significant drivers of asthma morbidity [3, 4], and exposure to these triggers is heavily influenced by social determinants of health [5–7], making asthma an important source of health disparities. Historically, community health worker (CHW) programs such as the Public Health-Seattle & King County (PHSKC) Asthma Program have brought CHWs into homes where they address environmental triggers directly by performing an environmental home inspection and provide education and support. The PHSKC Asthma Program and other similar programs have been consistently proven to improve asthma outcomes and reduce health care costs [8–14].

During the COVID-19 pandemic, in-home CHW services in the United States were significantly reduced or suspended, and many asthma programs transitioned their CHW services to remote or virtual platforms [15, 16]. However, virtual delivery of CHW services has not been well studied in terms of acceptability to families, accessibility, or effectiveness compared to in-person services. When considering a move toward virtual program delivery, CHW programs face two significant challenges. First, the use of video technology creates risk of widening the “digital divide,” referring to disparities in access to digital technology which are typically associated with socioeconomic factors and exacerbated by language barriers [17–20]. Secondly, an integral component of many in-home CHW services is the home inspection, in which environmental concerns are identified and addressed, which involves a careful in-person walkthrough of the home. Adaptation of this home evaluation to a virtual format seems challenging and likely to be less sensitive than an in-person walkthrough, and it is unclear if it would be as successful in improving health outcomes.

The aims of this study are to determine the perspectives of prior PHSKC Asthma Program clients on the acceptability and feasibility of a hypothetical virtual adaptation of the program, through the use of semi-structured interviews. We set out to include participants of all three languages served by the program: English, Somali, and Spanish. The results of this study are intended to inform the development of a virtual CHW programs that are both acceptable and feasible for families of children with asthma, and as a result more likely to be effective in improving asthma-related health outcomes.

## Methods

This is a qualitative study using semi-structured interviews to elicit the perspectives of participants. This research is conducted with a combination of deductive and inductive approaches. We focused on the concepts of acceptability and feasibility of a hypothetical virtual asthma program to structure our interview guide and analysis plan.

To define acceptability, we used the Theoretical Framework of Acceptability (TFA) of heath care interventions described by Sekhon et al., which represents a “multi-faceted construct that reflects the extent to which people delivering or receiving a health care intervention consider it to be appropriate, based on anticipated or experiential cognitive and emotional responses to the intervention” [21]. It comprises 7 mutually exclusive component constructs including affective attitude, burden, ethicality, intervention coherence, opportunity costs, perceived effectiveness, and self-efficacy.

We used the definition of feasibility of Proctor, et al. as “the extent to which a new treatment, or an innovation, can be successfully used or carried out within a given agency or setting” [22]. When considering an intervention from the perspective of the individual participant, feasibility can be conceptualized to include the technical competence of the individual and the availability of resources (further subdivided into availability of assistance, material resources, time, and financial resources to complete the intervention) [23].

We recruited prior clients of the Public Health-Seattle & King County (PHSKC) Asthma Program who had participated between Jan 2018-Feb 2020 (prior to the COVID-19 pandemic). These individuals by definition were adults aged 18 or older; were either an adult parent/guardian of a child with poorly controlled asthma (that is, asthma with frequent or disruptive symptoms), or had poorly controlled asthma themselves; and spoke either English, Somali, or Spanish. These individuals did not have experience with virtual visits for asthma. We used stratified purposeful sampling based on language in order to ensure representation of persons that are likely to face unique challenges in participating in virtual visits due to primarily speaking a language other than English. Equal importance was placed on participants speaking each language, but because the pool of Somali speaking individuals was far smaller than those speaking other languages, we were forced to adjust our target for this group down. Enrollment targets were 6–8 English-speaking participants, 6–8 Spanish-speaking participants, and 2–4 Somali-speaking participants, with total target of 20 participants. A total target of 20 participants was set as this was felt to be sufficient to reach saturation [24].

Participants were recruited via phone in their preferred language by bilingual research coordinators. The sole exclusion criterion was declining to be recorded. Prior to enrollment, informed consent was obtained and participants were informed about the research questions and the credentials and expertise of the primary researcher. Participants were provided a $75 gift card to a major grocery store chain as an incentive.

Semi-structured individual interviews were performed which included initial demographic questions followed by a series of open-ended questions. The interview guide was piloted among the study team of 4 people, including one research coordinator with personal experience with asthma, and was revised based on feedback. Interviews were performed in private either over the Zoom web application or via phone based on participant preference, in the preferred language of the participant by a bilingual research coordinator, who also recorded field notes. Each interview recording was transcribed by the same coordinator who conducted the interview, in the language in which it was performed. Interview transcripts in Spanish and Somali were then translated to English by a professional translation service. Transcripts were not shared with participants.

Data analysis was performed using a combination of deductive techniques (specifically framework-driven coding) and inductive techniques. Our *a priori* codebook used codes derived from the Theoretical Framework of Acceptability (affective attitude, burden, ethicality, intervention coherence, opportunity costs, perceived effectiveness, and self-efficacy) and the above definition of feasibility (technical competence, availability of assistance, material resources, time, and financial resources). In addition, we used codes to capture whether these constructs were discussed as a barrier/drawback to virtual delivery, versus a facilitator/benefit of virtual delivery.

ATLAS.ti (Version 23.2.1.26990) was used to manage and code data. Codebook development and the coding process was performed by the primary researcher, who was also the single coder (MEC). The first 5 interview transcripts were coded using the *a priori* codebook, and the codebook was modified and additional codes added as necessary. Subsequently, in order to establish intra-rater reliability, the single coder waited for a period of over one month prior to recoding the same 5 manuscripts, after which intra-rater reliability was determined by generating Krippendorff’s alpha [25] using ATLAS.ti.

Coding results were examined using code-documents tables, and code co-occurrence tables were used to analyze whether the constructs were viewed as barriers or facilitators and whether they differed between participants with different preferred languages. Finally, all suggestions for success of a virtual intervention were considered. These methods were used to develop themes to synthesize the data. There was no plan for participant checking or feedback. Methods and results were reported using the Consolidated Criteria for Reporting Qualitative Research (COREQ) Checklist [26], available in Supplementary Material 1.

Positionality: The study lead is author MEC, a pediatric pulmonologist physician and researcher with expertise in asthma. She is a white female cisgender person who has many years of experience in the clinical treatment of respiratory disease in underserved populations, who had no prior relationship with study participants. Due to concerns for differential in power and possibly outsider community status, Dr. Crocker did not perform any interviews personally. Study interviews were conducted by a team of research coordinators, including one who spoke English primarily, and two who were bilingual in English and either Spanish or Somali. One research coordinator had personal experience with asthma in a family member.

## Results

Interviews were conducted from December 2022 through February 2023. We contacted a total of 56 former clients of the PHSKC Asthma Program, and 24 of these agreed to participate and scheduled a study interview. Five of these could not later be contacted for the interview or changed their mind about participating. Ultimately, we interviewed a total of 19 participants (Table 1). Eighteen participants opted to complete the interview via Zoom, and one chose to be interviewed via telephone. Interviews ranged from 6 to 25 min in duration.

All 19 participants identified as female, despite this not being an inclusion criterion of either our study or the PHSKC program. Participant ages ranged from 37 to 65 years (mean 46 years). Nine participants spoke English, 8 spoke Spanish, and 2 spoke Somali as their preferred language. Most participants (95%) were parents of children with asthma as opposed to participating in the Asthma Program for only themselves (5%). The participants self-identified as Hispanic/Latina/Mexican (42%), Black/African American (37%), White/Caucasian (11%), Asian (5%), Hebrew (5%), or Native American (5%).

**Table 1:** Participant demographics

Characteristic	
Female, n (%)	19 (100%)
Age in years, mean (range)	46 (37–65)
**Preferred language, n (%)**	
English	9 (47%)
Somali	2 (11%)
Spanish	8 (42%)
**Who had asthma? n (%)**	
Client	1 (5%)
Child	17 (89%)
Client and child	1 (5%)
**Self-identified race/ethnicity**,^*^n (%)	
Asian	1 (5%)
Black or African American	7 (37%)
Hebrew	1 (5%)
Hispanic, Latina, or Mexican	8 (42%)
Native American	1 (5%)
White or Caucasian	2 (11%)

^*^This captures the participant’s response to the question, “What do you consider to be your race?” Some participants reported multiple racial/ethnic identities, leading to percentages totaling > 100%

After coding the first 5 transcripts (as above), Krippendorf’s alpha was computed to be 0.848, indicating high within-rater reliability. Code-document analysis showed that overall, participants offered many opinions on both benefits/facilitators and drawbacks/barriers of the hypothetical virtual asthma program, but comments trended more towards drawbacks (65 quotations regarding drawbacks versus 50 for benefits). This trend was also present when considering English and Spanish interviews separately, but Somali interviews tended to discuss more benefits/facilitators of a virtual model as opposed to drawbacks (13 versus 4 quotations). Further analysis allowed for the development of six common themes across participants, which are described in detail below along with supporting illustrative quotes.

**Theme 1. The face-to-face engagement between CHW and client and hands-on demonstrations are important for learning during the asthma home visit program, and would be diminished by virtual delivery.**

Participants expressed that the usual in-person format of the asthma program facilitated their learning through multiple mechanisms. Some suggested that topics that were more complex, requiring closer attention, or needing hands-on demonstration would be best performed in person (for example, health workers routinely provided education on how to hold a mask and spacer to use an inhaler, and this seemed challenging to accomplish over a video call). Many felt the face-to-face interaction helped them to stay focused on the material, and remain engaged with the CHW doing the teaching. Conversely, they felt that a virtual format would allow participants to not pay attention if less interested in the content.

*I don’t think that it should be completely virtual. There’s gonna be some demonstrations that have to be done in-person because with that [CHW], when she was teaching them how to use the spacers, she was allowing them to demonstrate to her how they did it, so she can see their technique. So that she can tell them whether it needed to be critiqued or whether it was properly used. I think that’s more of a hands-on thing than a virtual thing. ‘Cause everybody don’t get the technique virtually. They may need someone personally showing them how to hold the spacer, how to look for the little puff thing that puffs up and down that’s out of the spacer, how to count, I mean how to breathe it in all in, I mean those things can’t be done virtually and it’s breathing we’re talking about! (#4, English-speaking)*

*I have a learning disability too so I’m better with face-to-face so like I can get a better understanding… Yeah I learn better face-to-face, like hands on. (Participant #5, English-speaking)*

*But I think in person is ideal for me, because you learn more, right? In video calls you can talk and they can teach you, but to see the staff in person, in the moment, and them seeing things, I think that for me it would be much better in person. (#13, Spanish-speaking)*

A sense of personal connection or rapport was noted by several English- and Spanish-speaking participants to be important and was expected to be lost when participating remotely.

*[Virtual visits are] not too personable ‘cause you can talk to somebody, but actually showing them how to use it and being in their apartment so you can see the situation makes it more easier, for me that would be. Like you can walk around and show people [remotely], but they’re on camera, so do they really get a good view? Can they really see what’s happening? And then you meet the people, but you don’t get that real personal feeling and interaction you would if you would meet with somebody in-person. (#11, English-speaking)*

However, two other participants (speaking Spanish and Somali) presented the opposite opinion that this personal connection could be preserved by interacting with the CHW via video.

**Theme 2. Participants are concerned that a virtual adaptation of the home inspection would not be as effective as an in-person assessment.**

Participants identified the environmental home inspection as an integral part of the asthma home-visit program that was helpful for reducing asthma symptoms. During this component of the program, the CHW would perform a home walk-through, assessing for common issues likely to affect the client’s asthma (for example, mold, dust, or signs of pests). Many felt this helped them to identify issues in their homes, and thought the inspection would be essential to retain in a future version of the program. However, many (including 6 English- and 2 Spanish-speaking) participants expressed concern or doubts about whether a remote version of this assessment would be effective, and none suggested that a virtual adaptation would be sufficient or superior. It was unclear to some how such a remote assessment would work, and whether it could identify areas of concern with accuracy as well as an in-person evaluation. Participants worried that the CHWs would not be able to inspect the home closely, would be hindered by poor video resolution and glare, and would miss certain issues entirely.

*You can’t really do a home inspection through Zoom and I think that that’s important. I’m not sure how you’d get around that. Yeah I think that that would be the thing that would be pretty hard to manage from remote… You can give them the phone and tell them to walk around and show you stuff but, you know, that can only go so far… I think the most difficult thing would be that the community home worker can’t really see the environment which is super important for asthma management. (#2, English-speaking)*

*But I’ll be honest. I believe that in-person is more… you are seeing people’s needs. And obviously, right now I just put up a screen for you [behind where she is sitting], because I don’t want you to focus on what else is behind it, and you won’t know what else I have. I’m keeping my privacy. But when you have issues like asthma, I think it’s more than anything when you live in apartments. I think [in-person] would help more because you are seeing that there really is a problem. (#17, Spanish-speaking)*

**Theme 3. Convenience and flexibility are important benefits of virtual delivery of the home-visit program, but may be a double-edged sword.**

The convenience of scheduling and participating in virtual visits was frequently cited as a benefit of virtual delivery and an advantage over in-person. Some participants were attracted by the idea that they could be on the move when taking a video call, multi-task, or better fit a virtual call in between other obligations.

*A virtual visit would be better for me ‘cause I don’t always have time for people to come to my house and sit down. Like I’m always busy and on the move. And you can do virtual meetings on the move– you don’t have to be in one spot to do a virtual meeting… I can multitask. I can be out doing something else and logging on and taking that 15–20 minute break in between what I’m already doing to participate. (#4, English-speaking)*

Another participant acknowledged that virtual delivery might make things more convenient for the CHW as well, who would not need to travel for the visit.

*When they want to visit [in-person] they must allocate a time for you and drive a car to your place to meet you in person. But online is good, and easy for them, as they are in the comfort of their home, and it’s also easy for me. (#19, Somali-speaking)*

However, this convenience might have the unintended consequence of dividing the participant’s attention or preventing the CHW from assessing issues in the home remotely. One example of such a trade-off was evident when a research participant completed their Zoom interview for this study while they were walking through a shopping mall:

*I see some shoes—I’m about to buy me some tennis shoes. (#5, English-speaking)*

**Theme 4. Virtual visits were generally preferable to in-person in terms of privacy and safety, although they were not completely free of their own safety concerns.**

Privacy, safety, and moral appropriateness of the intervention were assessed through the concept of ethicality from the Theoretical Framework of Acceptability, which refers to whether an intervention fits within the individual’s value system. Several of our English- and Spanish-speaking participants volunteered that virtual visits felt safer due to concerns about getting sick with respiratory viruses if they participated in-person.

*Yes, virtual is a good alternative during COVID, and for safety and everything… We were afraid of COVID, it was worse before, but now it’s less so. It can be an opportunity for other children and other parents, who are afraid for their children, they’re parents who take care, because some children have more severe and strong asthma. (#25, Spanish-speaking)*

One participant suggested that in-person visits could be less acceptable to those who might be afraid of a government worker coming to their home, thinking they would be in danger of being arrested.

*We also have to know that some people don’t like to have visitors, they don’t want them to enter their homes. For example, if it is a state program they think “the police are going to take me in”, they think it is their fault. When that’s not true, right? And there are people who are scared when a worker arrives. I think [a virtual option] helps in many ways. It can be done virtually, individually or in groups. (#17, Spanish-speaking)*

On the other hand, virtual programs were not seen as free of their own safety issues, with one participant voicing concerns about scams and identity theft.

*With all the scams there are today, a lot of people are afraid to open a link. So I think a lot of people, and speaking with a lot of respect, I know they don’t like to search for anything on the internet. A lot of people don’t want to use it anymore. There are emails I don’t open anymore because maybe it’s a scam. This link sends me to this and that. You know that when you open something online, it doesn’t always open the right page, it tells you the second and third steps, and takes you where you need to go. Personally, I don’t like it much. (#17, Spanish-speaking)*

**Theme 5. Self-efficacy and competence with digital technology varied widely, even within language groups.**

Participants expressed a wide variety of comfort levels in using digital technology, and views did not vary predictably with the participant’s preferred language. Some members of all language groups expressed no difficulty with prior virtual experiences. Others described increasing comfort with the technology due to repeated use over the course of the pandemic. Still others described continued challenges, some of which were attributed more to the unpredictability of the technology or the user on the other end rather than their own lack of competence.

*Because when they [first] mentioned Zoom, I said: What is that? How do I get into it? It was like Chinese to me. But thank God we are moving forward. I think it’s nice to be on Zoom, it’s like being there in person and it’s helped us a lot. (#9, Spanish-speaking)*

*Well I’m a software developer so everything I do is virtual. I do doctor’s appointments, I work virtually, I do church virtual, I do Bible study virtual so I mean my life is virtual. (#12, English-speaking)*

*So, that’s why I’m telling you how difficult it is. It’s not like, for example, you send me a link now and I’ll see you right away. You see? It’s very different. But when you enter this number, and you do another thing, and you enter the code and all that, and you make mistakes because you don’t enter it right. If the call is cut off it is because a group I’m in is sending me messages right now. They’re telling me they can’t click a link, but I can’t answer right now because I’m in a meeting with you. I mean, can you imagine that they can’t click a link that was sent to them in the morning? [laughs]. (#17, Spanish-speaking)*

*There are people who can’t access Zoom online, people who don’t know anything about it, there are people who can’t use Zoom. Like those people, they would need to be visited at home because these people don’t know these things. For example, there are some uneducated mothers who can’t use Zoom…If they are told to access Zoom and they can’t use it or even maybe can’t dial a number, it might be difficult for them, do you understand? (#19, Somali-speaking)*

*It doesn’t always just be smooth-sailing. Sometimes you can’t get in and it’s not your fault, and you can’t let the person know that you can’t get in because they’re sitting waiting for you to come in so of course they know they have an appointment. So they’re not looking at their phones or they’re not trying to answer so they don’t know till you’ve already missed it. And it could be a very important appointment. And you missed it because you couldn’t log on. (#4, English-Speaking)*

**Theme 6. Concerns about internet connectivity were common.**

Many participants had concerns that their poor or inconsistent internet access would limit their ability to participate in virtual visits. One noted that connectivity problems increased when multiple children used the internet for school (a problem that was improving now that children were returning to school in-person).

*Logging on is sometimes a challenge because if you don’t log on in the proper spot or you don’t have the proper Internet connection, or you don’t have a wi-fi connection or you keep getting kicked out, I mean there’s a lot of factors that go on virtually. (#4, English-Speaking)*

*Well, my wi-fi sucks cuz I have–I’m on low-income internet, I only pay like $9 a month. So my wi-fi is really wishy-washy and then I have my oldest daughter on my wi-fi, I have my other daughter on my wi-fi, then I have my two youngest one on my wi-fi and then it’s just me and my husband on my wi-fi. So it gets messed up at times. (#5, English-speaking)*

*I don’t know why but just [region] itself has really bad Internet connection. Sometimes it’s very slow and then it just cuts off, and I don’t know if it has anything to do with the house across the street that has been torn down. I don’t know if it affected the telephone lines or the wire lines I don’t know… I know that [service provider] has been to this home I don’t even know, probably at least 10 times already in the last 12 months. (#10, English-speaking)*

In summary, participants revealed a great variety of perspectives regarding both the benefits and drawbacks of participating in a hypothetical virtual asthma program. Virtual participation was attractive due to convenience, flexibility, safety from respiratory viruses, and privacy. However, it was considered less ideal due to decreased learning or engagement, concerns about effectiveness of the environmental inspection, barriers to access such as lack of technical competence or internet connectivity, and perceived risk of scams.

## Discussion

In this qualitative study we interviewed former clients of the PHSKC Asthma Program, which uses the prototypical home visit model incorporating an environmental home assessment, asthma trigger mitigation, and education on asthma self-management strategies. This home visit model has been extensively studied and proven to be both effective and cost-saving in its classic in-home format. With the COVID pandemic many similar programs have transitioned toward a virtual approach. To our knowledge, this is the first study examining the perspectives of individuals or caregivers of children with asthma on virtual participation. Our results highlight a wide variety of perspectives regarding potential barriers and facilitators of virtual visits, as well as the benefits and drawbacks of in-person visits.

In general, our results showed that many participants felt positively about the prospect of completing the program virtually, and would appreciate being given this option. They expected virtual visits to offer the benefits of convenience, flexibility, privacy, and safety from exposure to respiratory viruses (as originally intended). The fact that interest in virtual visits was strong when these interviews were performed in early 2023 (when COVID transmission in King County was relatively low), suggests that there will continue to be demand for virtual programs in the future even when the health risk of in-person visits is low. This is consistent with other predictions that telemedicine is here to stay [27].

However, our results highlighted several major challenges with the acceptability and feasibility of virtual visits. First, it was clear that many participants highly valued the face-to-face interaction with the CHW in their home, suggesting that it boosted their engagement, helped them to learn, and simply felt more personal. They also reported benefitting greatly from hands-on demonstrations. This is not surprising, as engagement with the client is theorized to be a critical factor for the success of CHW programs [28]. Unfortunately, for some participants, that engagement is not as easily accomplished over virtual platforms. This may be exacerbated by the temptation for participants to multitask or commute during virtual appointments. Reduced engagement in the program is concerning as it could lead to diminished learning or lack of behavior change, rendering the program less effective. It is possible that this could be addressed through targeted efforts to increase rapport between CHWs and clients, limit distractions, and discourage multitasking.

A second major concern with a virtual adaptation relates to the perceived effectiveness of the environmental home inspection. Many participants felt the home inspection was an invaluable part of their participation in the in-person program, but they had difficulty envisioning how it would be performed remotely, and were skeptical that it could uncover issues in their homes with adequate sensitivity. This mirrors a knowledge gap that exists in the published literature: despite the fact that many programs have moved to virtual visits, it is not well documented what specific strategies programs are using to accomplish a remote home assessment, or whether a home assessment is being performed at all. More concerning, remote home assessment strategies have not been adequately evaluated for their effectiveness in uncovering environmental triggers or resulting in improvement in health. Because the home assessment and resulting recommendations for environmental mitigation strategies have been demonstrated to be essential to the success of the traditional asthma home visit model [29], it is critical to better understand whether a remote adaptation of this program component can retain its effectiveness. Additionally, our results suggest that even after this has been accomplished, remotely-based asthma programs may still have to work to overcome some skepticism in order to attract participants.

A final challenge highlighted by our results is the varying comfort levels with and access to the technology required to participate in a virtual program. Our study participants varied widely in their self-reported comfort level with digital technology and their access to devices and adequate internet connectivity. Even some who reported a high level of self-efficacy in using virtual platforms admitted that they still faced unexpected difficulties participating in virtual visits. Interestingly, these challenges were reported across all language groups and were not limited only to those speaking Spanish or Somali, and participants did not cite language barriers as a contributing factor. Addressing these technical challenges will require multiple strategies, such as providing a tutorial of the virtual platform in advance, real-time technical assistance, loaner devices, and wi-fi hotspots. This is especially important if programs are considering eliminating their in-person services in favor of virtual ones, in order to avoid paradoxically creating disparities in access to a program created to address social determinants of health disparities [18, 20].

Despite these recurring themes, each individual carried a unique combination of preferences and capabilities when it came to the idea of participating in a virtual visit for their asthma, and there seemed to be no “one-size-fits-all” approach. The ability to allow clients to choose between options of in-person or virtual may be ideal to increase access and comfort for all participants. As one participant stated, “I’m thinking that maybe if you guys have the option. Like if people were willing to let someone come in their home, I think it should be optional. Like they should be able to choose. If it’s a matter of comfort.” (#16, English-speaking) However, an important caveat to this is that maximizing acceptability and feasibility for clients in this way may come at the expense of other valuable benefits of the program, such as hands-on demonstrations and a thorough home inspection.

Another approach of great interest is to offer a hybrid program using both in-person and remote platforms at different times for the same client. The first visit could be performed in-person, providing an opportunity to develop rapport between the CHW and client, perform a high-quality in-person environmental home assessment, and provide hands-on demonstrations. Subsequent visits could be offered virtually (or the client could be given a choice between virtual or in-person), thus allowing the client to take advantage of the convenience, flexibility, and safety of virtual visits if so desired. However, to our knowledge, there is no published evidence comparing the effectiveness of various components of CHW asthma programs when delivered virtually versus in-person, so decisions regarding mode of delivery for each of these components must be made with caution, along with ongoing evaluation of program effectiveness where possible.

Based on the above considerations, we have compiled a list of recommendations to consider should programs choose to adapt their in-person asthma home visit programs to a virtual format (see Table 2). The next steps must include assessment of the effectiveness of a virtual adaptation of the home assessment, and of the virtual program in its entirety, ideally in comparison to an in-person strategy. Programs should document their strategies, successes, lessons learned, and evaluation results in order to advance knowledge in this area. Finally, in order to fully understand the benefits and drawbacks of a virtual model we must also evaluate the perspectives of CHWs on this modality and its cost effectiveness.

**Table 2:** Recommendations for adapting in-person asthma home visit programs to a virtual format

• If adapting the environmental home assessment to a virtual format, evaluate its effectiveness compared to in-person visits prior to full implementation.
• Encourage engagement during virtual sessions by making directed efforts to increase rapport between CHW and client.
• Discourage multitasking and travelling during virtual sessions.
• Find creative ways to perform virtual demonstrations of medications, equipment, and cleaning supplies.
• Provide real-time technical assistance with the virtual platform/software.
• Consider providing loaner devices (e.g. tablet) or wi-fi hotspot as needed.
• Consider offering both in-person and virtual options when safety/resources allow.
• Consider a hybrid approach (in-person home assessment and hands-on demonstrations, virtual follow-up sessions).

It is important to note that this study has focused on the acceptability and feasibility of virtual CHW asthma programs for participants, but there are a variety of other issues that should be taken into account when making decisions regarding mode of program delivery. As already discussed, program effectiveness in improving asthma control is an important consideration; in addition, program leaders may wish to evaluate cost, sustainability, ease of adoption, or feasibility on an organizational level. These concerns must all be weighed together, although we propose that high priority be placed on equitable access to effective services for the most vulnerable individuals.

Our study has several strengths. Firstly, we interviewed participants in three languages, with bilingual research coordinators using the language of the participants’ choice. Additionally, a diversity of races and ethnicities were represented, giving voice to a variety of groups. Our use of the Theoretical Framework of Acceptability and a previously established definition of feasibility strengthened our study design by giving a structure to the interview and subsequent analysis. The data were analyzed by a single coder, and intra-coder reliability was calculated to be high.

This study has several limitations which merit consideration. Our participants did not have prior experience with virtual visits for asthma, and thus we interviewed them about a hypothetical virtual program that they had not experienced; it is possible that their perspectives would be different had they participated in an actual virtual asthma program. However, they had all participated in the in-home PHSKC Asthma Program, and most had experience with virtual visits for other purposes, allowing them to offer valuable perspectives on our research questions. Another limitation is that we were only successful in recruiting a small number of Somali participants, due to the inability to contact the majority of the small number of eligible clients. Also, because we used three different interviewers, it is possible that there were inconsistencies in the depth or breadth of the interviews between languages, although we attempted to prevent this by using a common interview guide. Because the Somali and Spanish interviews required translation to English it is possible that some detail was lost in that process. Further, the coding and analysis were performed by a single analyst, which introduces the possibility of errors in interpretation. While we used determination of intra-coder reliability with Krippendorff’s alpha to ensure reliability, our study design would have been strengthened by using an second coder. Finally, the validity and confirmability of our results would have been enhanced by additional strategies such as member checks and auditing, but unfortunately resources did not allow for this.

## Conclusions

This study provides significant insight into factors that are very likely to influence the success of a virtual version of an asthma home-visit program. Virtual interactions are fast becoming the norm for healthcare, work, and school, and demand for virtual services is likely here to stay. In adapting asthma home-visit programs to a virtual format, it is essential to take the time to ensure the acceptability, feasibility, and efficacy of the adaptation to verify that it continues to provide the intended benefit without exacerbating existing health disparities. The recommendations provided in this paper serve as a starting point to enhance future efforts to make such adaptations.

### Electronic supplementary material

Below is the link to the electronic supplementary material.

**Supplementary Material 1**: COREQ 32–Item checklist

## Data Availability

The datasets generated and analyzed during the current study are not publicly available to protect the privacy of participants and in accordance with our institutional review board approval. If you would like to request data from this study, please contact the corresponding author (Dr. Mary Crocker).

## Abbreviations

CHW: Community health worker
COREQ: Consolidated Criteria for Reporting Qualitative Research
COVID-19: Coronavirus disease 2019
PHSKC: Public Health—Seattle & King County
TFA: Theoretical Framework of Acceptability
