# Supplementary material for: A qualitative study of perspectives on the acceptability and feasibility of “virtual home visits” for asthma
Source: BMC Public Health. 2023 Dec 20;23:2546. doi: 10.1186/s12889-023-17485-8 (PMC10734151; doi:10.1186/s12889-023-17485-8)
Supplement: Supplementary file 1 — Supplementary Material 1: COREQ 32–Item checklist [file 12889_2023_17485_MOESM1_ESM.docx]

**COREQ 32-­‐ITEM CHECKLIST**

**for**

A qualitative study of perspectives on the acceptability and feasibility of “virtual home visits” for asthma

| **Item** | **Guide questions/description** | **Section** |
| --- | --- | --- |
| Domain 1: Research team and reﬂexivity | | |
| 1. Interviewer/facilitator | Which author/s conducted the interviews or FGDs? | Methods |
| 2. Credentials | What were/are the researcher’s credentials? | Author information, Methods |
| 3. Occupation | What was their occupation at the time of the study? | Author information, Methods |
| 4. Gender | Was the researcher male or female? | Methods |
| 5. Experience and training | What experience or training did the researcher have? | Methods |
| 6. Relationship with participants established | Was a relationship with participants established prior to study commencement? | Methods |
| 7. Participant knowledge of the interviewer | What did the participants know about the researcher? | Methods |
| 8. Interviewer characteristics | What characteristics were reported about the interviewer/facilitator? | Methods |
| Domain 2: study design | | |
| 9. Methodological orientation and Theory | What methodological orientation was stated to underpin the study? | Methods |
| 10. Sampling | How were participants or articles selected? | Methods |
| 11. Method of approach | How were participants approached? | Methods |
| 12. Sample size | How many participants or articles were in the study? | Results |
| 13. Non-­‐participation | How many people refused to participate or dropped out, or articles were excluded during review? For what reasons? | Results |
| 14. Setting of data collection | Where was the data collected or what regions of articles are represented? | Results |
| 15. Presence of non-­‐ participants | Was anyone else present besides the participants and researchers? | Methods |
| 16. Description of sample | What are the important characteristics of the sample? | Results |
| 17. Interview guide or alternative data collection tool | Were examples from questions, prompts, guides provided by the authors? | Available upon request |
| 18. Repeat interviews | Were repeat interviews carried out, or were multiple articles from the same study reviewed? | Methods |
| 19. Audio/visual recording | Did the research use audio or visual recording to collect the data? | Methods |
| 20. Field notes | Were ﬁeld notes made during and/or after the interview/FGD or article review? | Methods |
| 21. Duration/Length | What was the duration of the interviews/FGDs or length of the articles? | Results |
| 22. Data saturation | Was data saturation discussed? | Methods |
| 23. Transcripts returned | Were transcripts returned to participants for comment and/or correction? | Methods |
| **Domain 3: analysis and ﬁndings** | | |
| 24. Number of data coders | How many coders helped code the data? | Methods |
| 25. Description of the coding tree | Did authors provide a description of the coding tree (ie: codebook)? | *A priori* codes described in Methods; full codebook available upon request |
| 26. Derivation of themes | Were codes/categories identiﬁed in advance (deductive) or derived from the data (inductive)? | Methods |
| 27. Software | What software, if applicable, was used to manage the data? | Methods |
| 28. Participant checking | Did, or will, participants or article authors provide feedback on the ﬁndings/results? | Methods |
| 29. Quotations presented | Were participant quotations or article text presented to illustrate the themes/ﬁndings/results? Was each quotation identiﬁed? | Results |
| 30. Data and ﬁndings consistent | Was there consistency between the data presented and the identified themes? | Results |
| 31. Clarity of major themes | Were major themes clearly presented in the ﬁndings? | Results |
| 32. Clarity of minor themes | Is there a description of diverse cases or discussion of minor themes? | Results |
